# What Do White Parents Teach Youth About Race? Qualitative Examination of White Racial Socialization

**DOI:** 10.1111/jora.12782

**Published:** 2022-07-12

**Authors:** McKenna Freeman, Andrew Martinez, Vaishali V. Raval

**Affiliations:** ^1^ Miami University

**Keywords:** White racial socialization, White privilege, race, racial oppression

## Abstract

Research has examined racial socialization practices within families of color, but less is known regarding what White parents teach their children about race and/or racism. To explore White racial socialization processes, we interviewed 30 White parents of White children ages 7–17 years living in the Midwest. Using thematic analysis, we identified 22 themes organized into four domains: Content of conversations, factors to consider in socialization, developmental differences, and White identity/privilege. A majority of parents reported conversations about current or historic racial events, while relatively few also reported speaking specifically about systemic racism and microaggressions. Parents viewed adolescents as better able to handle difficult topics than children. Findings contribute to theoretical frameworks and may inform the development of educational resources.

There is growing literature on how parents of color discuss racialized experiences with their children and prepare them for experiences of racism, a process referred to as ethnic racial socialization (ERS; Hagerman, 2017; Umaña‐Taylor & Hill, 2020). However, relative to this work, much less is known about what White children and adolescents learn about race and racism in their families while growing up, referred to as White racial socialization (WRS; Abaied & Perry, 2021; Loyd & Gaither, 2018; Underhill, 2018). Research pertaining to White parent–child conversations about race is critical because the types of conversations in the family of origin (or lack thereof) may contribute to further oppression of people of color, or to better understanding of systems of oppression and privilege and promote potential allyship. The purpose of this qualitative study was to explore the perspectives of White American parents about whether they have conversations with their children about race, their rationale for their decisions, the content of those conversations, and differences in types of conversations across child age. Exploring White parents' racial socialization practices within the current socio‐political context is critical due to increased public awareness of social justice movements (e.g., Black Lives Matter, Stop Asian Hate) and increased tension in interracial relations.

## Racial Socialization in White Families

Ethnic racial socialization has been defined as a social, cognitive, and developmental process in which ideas, beliefs, values, social norms, and behaviors surrounding race and ethnicity are transmitted, interpreted, negotiated, and adopted (Loyd & Gaither, 2018). ERS varies in both the process and the content of these messages (Hughes et al., 2006; Neblett, Rivas‐Drake, & Umaña‐Taylor, 2012). Messages come from multiple sources including parents, peers, teachers, and media, and may include either explicit or direct conversations and implicit messages or observations (Loyd & Gaither, 2018).

The construct of ERS was initially identified and studied in the context of families of color to understand how parents pass along cultural values and socialize their children to function in mainstream White society by preparing them for experiences of racial discrimination (Hughes et al., 2006). Research has shown that racial socialization occurs less frequently in White families compared to families of color (e.g., Hughes et al., 2006) and White individuals are less likely to think about themselves in terms of race (e.g., Herman, 2004; Lewis, 2004). Some White parents may not believe race is an important topic to discuss with their children (Katz, 2003), whereas others consider it important but do not engage in such discussions (Vittrup, 2018). White parents' decision to not talk about race, racism, or their White identity is a form of racial socialization that undermines the role of race.

Scholars have defined color‐blind racial attitudes as attitudes that contribute to minimizing or denying differences, emphasize the idea of equal opportunities, and support the idea that prejudice or discrimination will end if it is not confronted or discussed (Bonilla‐Silva, 1997; Edwards, 2017; Neville, Awad, Brooks, Flores, & Bluemel, 2013). Individuals who endorse these attitudes minimize the experiences of people of color and these attitudes have been classified as a racial microaggression (Sue & Spanierman, 2020). Additionally, these attitudes have also been characterized by two domains including color‐evasion (denial of racial differences) and power‐evasion (denial of racism by emphasizing equal opportunities; Neville et al., 2013). Overall, individuals who hold these attitudes deny the role that systemic racism has played throughout history in the structural organization of the United States, including the consequences of colonization, dynamics of political rule, aftermath of slavery on the labor system and economy (Bonilla‐Silva, 1997; Feagin, 2013). Some evidence indicates that individuals who hold color‐blind racial attitudes have increased racial biases, however, more work is needed to determine factors that motivate these approaches and the associated outcomes (Neville et al., 2013; Pahlke, Bigler, & Suizzo, 2012).

Apart from not talking about race or emphasizing color‐blind racial ideology in one's socialization efforts, some White parents emphasize color‐conscious approaches in their socialization that are centered on the acknowledgment of differences in experiences based on race. A review of literature on WRS summarizes White parents' approaches as either racially color‐blind or color‐ conscious (Loyd & Gaither, 2018). However, research has shown that some White parents use a combination of racially color‐blind and color‐conscious approaches (Abaied & Perry, 2021).

Recent social justice movements (i.e., Black Lives Matter), increased hate crimes toward Asian Americans during COVID‐19 (Tessler, Choi, & Kao, 2020), and political debates related to immigration policies and the US–Mexico border security have increased the public conversation surrounding race relations in the United States. Within this changing socio‐political context and visibility of the racism pandemic, it remains unclear whether and how White parents are discussing these events with their children and talking about broader systems of oppression. Further, WRS messages and practices may be more nuanced beyond a racially color‐blind versus color‐conscious categorization, and may include a more complex set of messages that have not yet been identified.

## Understanding of Race Across Childhood and Adolescence

Children's understanding of race develops through their participation in mainstream society, often with little direct socialization from their parents (Quintana & McKown, 2008). Research suggests that children perceive visible markers of racial identity (e.g., skin color, hair texture) as early as age 2 (Nesdale, 2013), and may begin to show racial biases by age 4, such as showing preferences for individuals from the majority group (Bigler & Liben, 2007). Additionally, children at this age are able to comprehend and endorse stereotypes (Bigler & Liben, 2007). However, 60% of White parents of kindergarten‐age children reported never talking with their children about race (Lesane‐Brown, Spatzier, & Tobin, 2010), and of those who did, 70% of White mothers reported using racially color‐blind approaches in their socialization (Vittrup, 2018). The desire to not talk about race may be due to the belief that discussions about race may make their children more prejudiced if the child is too young (Vittrup, 2018). However, research suggests that color‐conscious discussions surrounding race can reduce stereotyping and increase detections of racial bias in children (Bigler & Wright, 2014; Johnson, Rush, & Feagin, 2000). Nonetheless, the limited research suggests that although White children notice markers of race, many White parents choose to not engage in discussions surrounding race at this age.

By age 6–7, children are able to understand the concept of ethnicity (Quintana & McKown, 2008) and by age 10, both White children and children of color may be able to interpret situational cues in interracial situations and understand discrimination (Verkuyten, Kinket, & van der Wielen, 1997). Interestingly, White parents' preferences about whether to discuss these racial issues with their children vary. For example, in one study, most parents of White children at this age expressed color‐blind racial ideology and were reluctant to mention how racism and stereotypes may impact their children's interracial friendships (Hunter, Friend, Williams‐Wheeler, & Fletcher, 2012). Another study found that even in situations where racial bias is salient and explicit, White parents were unlikely to discuss racism explicitly (Zucker & Patterson, 2018). White parents who held less biased racial attitudes were more likely to engage in color‐conscious as opposed to color‐blind racial socialization, and were more likely to discuss racial issues with their children (Zucker & Patterson, 2018).

In early to late adolescence, parents often rely on the children's schools and peers to teach their children about race (Hamm, 2001). Some White parents have reported choosing to send their middle‐school age children to racially diverse schools in order to have increased contact with diverse groups of peers (Hagerman, 2014). However, solely relying on the presence of diversity is likely not enough to create a positive impact on children's interracial beliefs (e.g., Aboud, Mendelson, & Purdy, 2003; Quintana, 1998). For example, a child may have a negative interaction with an individual from another racial group, and may transfer that negative attitude to others of that racial group (Quintana, 1998). However, explicit conversations with parents can help White children to process these experiences and avoid generalizing interactions with individuals to entire groups. For example, White children who received lessons on historical racism toward African Americans showed less bias toward African Americans (Hughes, Bigler, & Levy, 2007).

The developmental period of adolescence is marked by increased independence and peer influence. Unlike the previous stages, adolescents are more likely to form personal opinions, including racial attitudes, which may deviate from their parents' beliefs (Hagerman, 2014; Steinberg & Morris, 2001). Because of increased access to technology and social media, adolescents today have easier access to social movements, information about publicized events, and different opinions than previous generations. This increased access may motivate adolescents to initiate conversations and discuss these topics with their parents. The ways in which parents approach WRS or respond to adolescent‐initiated conversations and discuss topics related to race may have implications for adolescents' developing understanding of the experiences of people of color and of interracial relations. Although literature on WRS is limited, it is particularly scarce during the developmental period of adolescence (Loyd & Gaither, 2018).

## Current Study

Our aim was to explore the perspectives of White American parents regarding whether they have conversations regarding race and racism with their White children and adolescents (ages 7–17 years), and why/how they approach the topic. Qualitative research methods are particularly well‐suited to this inquiry as they allow us to understand parent–child conversations from parents' perspectives, explore beliefs, and motives guiding parents' decisions in an open‐ended manner, and help identify the content of these conversations. In a semi‐structured interview, the participant can discuss their beliefs and provide responses in their own words rather than rate their agreement with statements provided by the researcher (Lord, Schnarr, & Hutchison, 1987).

Using thematic analysis (Braun & Clarke, 2006) that acknowledges researcher subjectivity and participants' experiences as contributing to the research process (Morrow, 2005), we addressed the following research questions: (1) Do White parents talk to their children about race and/or racism? If so, what is the content of these conversations? (2) What factors do parents consider when deciding to have or not have these conversations? (3) How do parental conversations about race and/or racism differ based on the child's age and developmental period? And (4) How do White parents talk about White identity?

We focused on White parents who had White children between the ages of 7 and 17 years. We decided to include children as young as 7 years old because around age 6 to 7, children not only attend to visible markers of racial differences, but also understand concepts such as ethnicity (Quintana, 1998) and fairness, and strive to appear fair in their actions (Shaw et al., 2014). We chose the upper age limit for inclusion as 17 years due to particularly limited literature on WRS among White adolescents. Our goal was to compare WRS themes between parents of children aged 7 to 11 years and 12 to 17 years to examine similarities and differences in the frequency and type of themes (e.g., whether parents of 12‐ to 17‐year‐olds engage in different types of conversations compared to 7‐ to 11‐year‐olds).

## METHOD

### Participants

Participants included 30 White parents of White children in the United States (*M*_age_ = 40.83 years, *SD* = 5.47, 96.7% mothers). Inclusion criteria included White Americans who reside in the United States, speak fluent English, and have at least one White child between the ages 7 and 17 years. Specifically, 40% had one or more children between 7 and 11 years of age, 36.7% had one or more children between 12 and 17 years of age, and 23.3% had a child in both of these age groups. Overall, a majority of the participants reported having a total of two or more children (83.3%). Additionally, two participants reported also having a non‐White adopted child. Most participants (50%) identified as middle class, 30% as upper middle class, 16.7% as lower middle class, and 3.3% as working class. Regarding yearly income, 6.7% reported household yearly income between $25,000 and $50,000, 43.3% of participants reported between $50,000 and $100,000, 40% reported between $100,000 and $200,000, and 10% reported above $200,000. The majority of participants reported currently residing in Ohio (96.7%) and reported varying education levels: 10% some college, 3.3% 2‐year degree, 33.3% 4‐year degree, 43.3% Master's degree, and 10% Doctorate or other professional degree. The majority of participating parents (83.3%) were married.

### Procedure, Data Sources, and Researcher Positionality

As recommended by Levitt et al. (2018), we describe below the context of the data sources (when and where data were collected) as well as the context of researchers. This study was approved by the institutional review board of the first author's academic institution. Participants were recruited through electronic fliers posted in various community, childcare, and parenting Facebook groups in the Midwest. Interested parents were encouraged to contact the researchers via email and then completed an online consent and demographic form (to gather information about parent gender, age, education level, profession, relationship status, number and age of children, and SES). All interviews were conducted virtually using WebEx between September 2020 and January 2021 and were digitally audio recorded. Interviews were transcribed and transcripts were uploaded into Dedoose, a mixed‐methods data analysis software.

All interviews were conducted by the first author, who is a White Mexican American cisgender woman graduate student. The author's visible social identities (name and physical appearance as a White woman) may have facilitated White parents feeling more comfortable discussing race‐related issues as she was likely perceived as an in‐group member. However, the author's position as a graduate student in an academic setting may have encouraged responses motivated by social desirability. Additionally, the first author's personal experiences of being raised by one White parent and one Mexican parent allowed her the opportunity to understand participants' experiences from both an insider and an outsider perspective. The second author, an undergraduate student who is a White Belizean American cisgender man (primarily identifies as White), assisted with transcribing interviews and coding data to establish interrater agreement. The third author, an Asian American cisgender woman faculty member, met regularly with the first author to discuss data themes and organization. Based on the existing literature, the authors' expectations were that most White parents do not engage in frequent, specific conversations about race and racism with their children. To manage researcher perspectives during data collection, the first author worked to monitor her reactions during the interview to maintain neutral face and tone, recording personal notes after each interview, and frequently meeting with other authors to discuss the process. However, it is important to note that qualitative data are co‐constructed through conversations between the interviewer and the interviewee, and thus, represent co‐construction of participants' experiences and perspectives.

#### Open‐ended interview protocol

A semi‐structured interview protocol was developed to target parental beliefs and strategies surrounding WRS that included a total of 22 questions. The interview protocol began with broad questions about whether parents talk to their children about race and ethnicity, as well as their rationale for their decision to talk or not talk about these topics. Parents were asked about the kinds of things they discuss with their child (if they report having these conversations), whether children have initiated such conversations, and how parents have responded. Parents were also asked whether they have talked to their children about poor treatment of people of color, with specific prompts about deaths of Black Americans due to police brutality, family separation at the US–Mexico border, Islamophobia in the United States since 9/11, and increased hate crimes toward Asian Americans during COVID‐19. After the interview, participants were sent an optional follow‐up survey about the interview, and participant responses indicated an overall positive interview experience.

### Strategy for Data Analysis and Establishing Trustworthiness

Following Morrow's (2005) guidelines for establishing trustworthiness in qualitative research, we used an approach in which the truth is relative and the interviewer and participant both bring their individual experiences and perspectives to the interview. Interview transcripts were analyzed using six‐phase thematic analysis (Braun & Clarke, 2006), which includes familiarizing oneself with the data, generating initial codes, searching for themes, reviewing potential themes, defining and naming themes, and producing the report. The first author conducted all interviews and read through all transcriptions to familiarize herself with the data. Subsequently, she created a code book with descriptions and examples of each code. Then, she used the final code book to code all interviews. Data saturation was reached by the 22nd interview such that no new themes were coded beyond this interview. To establish credibility, the first author regularly met with the third author, who has expertise in qualitative research and literature concerning privilege and marginalization in the United States, to help uncover and address biases. Following the guidelines by O'Connor and Joffe (2020), the second author was trained and coded 1/3 of the interviews to establish inter‐coder agreement. The first and second authors met regularly to review coding and discuss any areas of disagreement in codes. Cohen's Kappa across codes ranged from .80 to 1.0 indicating substantial agreement. To ensure data integrity, the first author frequently asked follow‐up questions during the interview to clarify what participants said or meant as needed.

## FINDINGS

Based on thematic analysis (Braun & Clarke, 2006), we identified 22 themes that are arranged in broader domains to address our research questions of: (1) Do White parents talk to their children about race/racism? If so, what is the content of these conversations? (2) What factors do parents consider when deciding to have or not have these conversations? (3) How do parental conversations about race/racism differ based on the child's age and developmental period? And (4) How do White parents talk about White identity?

### Domain One: Content of Conversations

**FIGURE 1:**
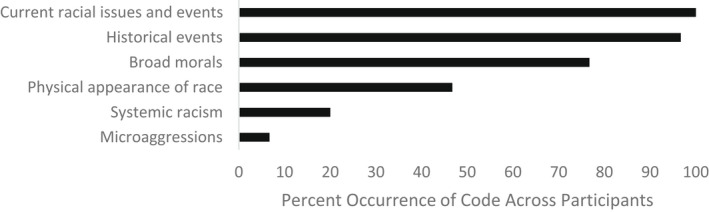
Domain I: Content of conversations.

When asked whether they talk to their children about race, all parents in our sample reported that they do, though we did not ask about how frequently they have these conversations. Parents identified a variety of topics of race‐related conversations that were coded into six themes including discussions of current racial issues and events, historical events, broad morals, physical aspects of race, systemic racism, and microaggressions (see Figure 1).

#### Current racial issues and events

Parents reported discussing a variety of current events related to race with their children including discussions of Black Lives Matter, police issues, discrimination against Asian individuals during COVID, and immigration. Some conversations focused on the differences between “Black Lives Matter” and “All Lives Matter” and why there is a movement that specifically focuses on Black lives. For example, one parent stated “…he [son] would've of course see[n] things…with the whole ‘oh well all lives matter’. And I would say to him ‘right but… all lives aren't in trouble right now.’”

Interestingly, although all parents expressed support for BLM, some parents went on to condemn rioting and disagreed with acts of violence, emphasizing the need for peaceful protesting. For example, “I believe that there are people that are peacefully protesting… and they are just trying to get their voices heard… and there are crazy people in every situation.” Similarly, after expressing support for BLM, some parents went on to talk about respect for the police and seeing “both sides.” For example, one parent stated, “…it breaks my heart because I would say almost every police officer I know is a good person and does a great job.” Another parent said,

…my dad is a retired (City) police officer and… she [daughter] said to him [daughter's grandfather]… “Did you ever do that papa?” and he was like, “No… There's good and bad anywhere you go. There's good doctors and bad doctors and if you notice something that isn't right then you should tell somebody.”

Additionally, few parents reported not publicly expressing support for BLM due to not wanting to upset friends or family who are police officers. For example, one parent stated, “My stance is that like I wouldn't put any of those signs in my yard because I don't… I don't want one group of people to feel like I'm against them.”

In addition to BLM, some parents reported talking with their children about discrimination targeting Asian/Asian American individuals during COVID. One parent stated, “…we were talking about COVID and how Americans were looking at Asians as the reason we had COVID‐19 here. I did relate it back to after September 11th… and even now how… Islamic or Muslim families were discriminated against.” Another parent reported “…talking about that people from China are not bad because the virus may or may not have originated in China…And you know that… it's never okay to you know hate an entire group of people.”

Finally, there were references to conversations surrounding immigration including topics such as the U.S.–Mexico border/the wall, refugees, and adoption. For example, one mother of a White child and an adopted child from an Asian country reported having conversations with her White child regarding anti‐immigrant sentiment overheard from peers at school. Additionally, several parents also reported discussing children being separated from their families at the U.S.–Mexico border, as well as discussing acceptance of peers who do not speak English as a first language.

#### Historical events

Although parents referenced taking about historic events almost as frequently as current events, their narratives regarding conversations about historic events involved a few well‐known issues and movements in history (e.g., slavery, civil rights movement, Native American struggle), and were relatively less elaborate (e.g., saying that they talked about slavery but not describing in‐depth what they talked about) than narratives for current events. For example, one parent shared, “We've talked about Native Americans… all the different battle sites around here and… talked about how devastating it was to… have their land taken.” References were also made to visiting museums or memorials. Other parents discussed what the children were learning in school. For example, one parent reported,

I actually made sure… that they weren't being lied to (laughs)… “So you learned about Thanksgiving. What did you learn about Thanksgiving?” … as they got older… “Did you learn about the trail of tears? Did you learn about relocation? Did you learn about… the genocide of the Native American people?”

Importantly, this explicit attempt to discuss historical oppression in the context of school lessons was rare in this sample, and most parents referenced engaging in conversations about historical events in general without specifically challenging what their children were learning in school.

#### Broad Morals: “Treat everyone equal/with respect”

These responses focused on broad conversations such as treating everyone as you would like to be treated, treat everyone with kindness and respect, and general bullying concerns without an explicit reference to race. For example, “…we just talk about you know treating others with kindness and treating others equally, but I do not like necessarily bring up like a certain race… we just talk about being kind to everyone that we interact with.” When discussing bullying, one parent stated, “I think we just try and teach values of loving other people but we don't always specify certain groups.”

#### Physical appearance of race

Some parents mentioned discussing the genetic or evolutionary aspect of skin color, including the concept of melanin, ancestry, and multiracial individuals. For example, “…I remember having conversations with him about…melanin and how skin color is different and it depends where your ancestors come from…” Additionally, some parents shared that their children asked questions about peers who had different racial identities from their parents or siblings, which led to conversations about family structures, adoption, or multiracial individuals, and families where parents explained why a child's physical appearance (e.g., skin tone) is different from one or both parents.

#### Systemic racism

Relatively few parents reported specifically talking or planning to talk with their children about the concept of systemic racism. One parent stated,

I want to teach them about the systemic racism… the fact that… we don't all begin our lives… on the same starting line with the same privileges and that there's… 400 years of history of why things are the way that they are now and so to… dig deeper and to recognize um the inherent privilege that they have…

Another parent identified their own increased awareness of systemic racism during the COVID‐19 pandemic and having discussions with their child about the disproportionate impact of the pandemic on different racial/ethnic groups. A third parent reported having general discussions about the existence of systemic racism, including how White individuals benefit from systems which oppress people of color.

#### Racial microaggressions

Very few parents mentioned specifically discussing racial microaggressions with their children. For example, one parent shared “…we tried to help him understand when somebody says a microaggression, sometimes they don't mean to hurt anybody, it's just they haven't thought about their bias.” Here, the parent is explaining microaggressions as unintentional acts that are a result of being unaware of one's biases.

### Domain Two: Factors to Consider in Socialization Practices

**FIGURE 2:**
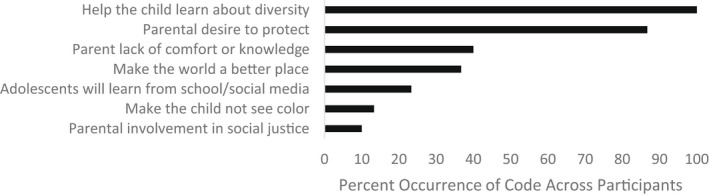
Domain II: Factors to consider in socialization processes.

The second research question addressed what factors parents consider when deciding to have these conversations and how to approach them, including both potential benefits and challenges. Parents identified several important factors which inform their socialization practices which were coded into seven themes, including concerns that were child‐focused and those that were parent‐focused. In order of most to least frequent, these factors included a desire to help their child learn about diversity (child‐focused), to protect the child (child‐focused), parents' lack of comfort or knowledge (parent‐focused), parents' desire to make the world a “better place” (parent‐focused), the likelihood of their child learning about these topics elsewhere (child‐focused), make the child not see color (child‐focused), and parents' own involvement in social justice activities (parent‐focused) (see Figure 2).

#### Help the child learn about diversity

All parents identified conversations about race as benefitting their child in learning about diversity. For example, some parents focused on the importance of conversations about race for the child's education, such as educating about current injustices or historical events. For example, “I just want them to realize there's a problem in this country and that they should always act to make sure that… when they're confronted with racism, that they address it.”

Parents also focused on the importance of helping children to think critically and independently, form their own opinions, and use reliable sources of information. For example, “I like hearing his point of view on it. I like seeing how he feels about different subjects and where he stands and why. I like him to have backing to his sources and any claims that he would have.”

Finally, some parents also focused on teaching children to notice, appreciate, and celebrate differences regarding race. For example, “…our goal is to make sure he was never color blind and that's really what we talked about most with him when he was younger. And seeing color and seeing beauty in color.”

#### Parental desire to protect

A majority of parents endorsed concerns regarding protecting their children from potential harmful or uncomfortable effects following conversations about racial issues. First, some parents reported not engaging in conversations about race or limiting engagement based on the child's age and maturity level, and this was more common among parents of 7‐ to 11‐year‐olds (42% vs. 36% among parents of teens). For example,

… I might talk about with my 11 year [old]… “there was an instance where somebody was… mistreated and it happened to be a Black person and a White police officer.” … With my 7‐year‐old I… wouldn't talk about that specific incident because he wouldn't understand. He might think… “Oh so police officers are not safe” (laughs) and I wouldn't want him to think that.

Another variable that some parents considered is child characteristics or personality, such as being an anxious or a sensitive child, and this was more common among parents of teens (56% vs. 33% among parents of younger children). As one parent stated, “I would worry how that would affect them um especially my 13‐year‐old, he feels everything so deeply” and as another illustrated, “…unfortunately, he is a super super anxious kid… I just don't know that he can handle some of the… facts about those types of cases, right?” Additionally, some parents, particularly parents of 7–11‐year‐olds, explicitly endorsed concerns over not wanting to scare their children during these conversations, such as the possibility of instilling fear of violence at protests or fear of police officers.

A third concern of parents involved fears that the child would overemphasize the importance of race, or the child may begin thinking about race if they were not already. For example, “I don't want to bring it to their attention if to them there's no difference with any of their friends, right?” Parents expressed a desire to be positive and not focus on the negative when having conversations about race. For example, “sometimes I wonder if I've shared too much um negativity when they should just be a kid and be playing in piles of dirt.” Another parent reported emphasizing the progress of racial justice in the United States and “focusing more on the positives of where we've come,” for example, “to the point where we can be best friends and we can ride the same bus and we can go to the same school and they sit right next to you.”

#### Parents' lack of comfort or knowledge

Some parents identified their own lack of knowledge about these topics as a challenge when having conversations about race. For example, “I think one of my biggest… concerns is just my relative lack of experience…my intentions are good. I don't always understand the issues, at least the core of the issues that people are talking about.”

#### Parent desire to make the world a better place

These responses primarily focused on having conversations with children in order to make the world a better place. For example, “…the world would be a better place… this kind of thing [racism] would not be seen… even specific things like police officers would be better vetted before joining the police force… and… bigger things like our country as a whole.”

#### Adolescents will learn from school/social media

In contrast to parents who discussed factors leading them to not engage in conversations about race, other parents, in particular, parents of adolescents emphasized the fact that adolescents will hear about these issues at school or from social media, and thus, it is important to discuss them. For example, in reference to the death of George Floyd, one parent explained, “… some of them [adolescents] are on Instagram and Snapchat… and they're not going to be able to hide from it [news about police brutality].” Due to this access to social media, some parents also shared that their adolescents were initiating conversations about race and racial justice with them.

#### Parental desire to make the child not see color

Although relatively infrequent, a few parent responses focused on color‐blind racial socialization approaches of wanting children to not see or acknowledge race in other individuals. For example, “I hope they don't see color. I hope they just see a person as their friend” or “…I want them to have the whole color‐blind thing when…interacting with people of other ethnicities and races.”

#### Parental involvement in social justice activities

A small subset of parents reported that their own parents spoke to them about these topics, or that they themselves were involved in activism and social justice, which aided them in having these conversations with their children. For example, “… I tell my kids… I grew up having these conversations around the dinner table… about racial injustice, so they know that I grew up also in a family that was concerned about those kinds of issues.” Another parent explained, “We attend… a unitarian community… and I sit with the social justice committee there and so… they [child] are very aware of you know what's going on socially… it's something that we discussed like on a regular basis….”

### Domain Three: Developmental Differences

**FIGURE 3:**
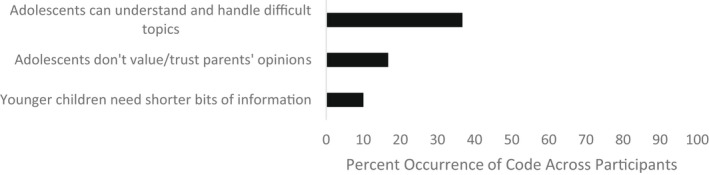
Domain III: Developmental differences.

Parents discussed developmental differences in how they approach these conversations with younger versus older children and responses were coded into three themes. Parents reported that older children can understand and handle difficult topics, adolescents do not value their parents' opinions in the same way that children do, and younger children need shorter conversations (see Figure 3).

#### Adolescents can understand and handle difficult topics

Many parents reported discussing difficult topics with their adolescents compared to children. For example,

…when she was in grade school, we'd have a lot of conversations about non‐violent things basically…and I didn't have a conversation about “gee and a new person got beat up today or a new person got murdered today.” I mean that's certainly not the conversation I had with her in grade school or even in middle school, that was more that was a more recent development as she developed her own activist life basically, in high school.

A parent with a younger child reported,

I think it's super important that they learn about civil rights and I think he's old enough to know about that now… I think I would tend to teach the historical aspects um of racism and slavery and civil rights and Native Americans…for the first few years and then talk about like what's happening more today like later…maybe like 10ish.

There was some variation in beliefs about the age that is appropriate for having difficult conversations. For example, one parent reported that her 7‐year‐old was old enough to understand concepts such as oppression and police brutality, while another parent reported that her 12‐year‐old was not yet ready for such conversations.

#### Adolescents do not value/trust parents' opinions as much as children

Some parents reported their adolescents not engaging in conversations and parental beliefs that adolescents do not value or trust their parents' opinions. Other parents reported fears that this would happen as their children enter adolescence. For example,

…right now I could say something and they would be like “oh yeah, yeah mom.” But I'm sure as they get older they'll be like “well mom I don't know”, because they're going to form their own opinions and they're…probably not going to take everything I say at face value as they do at this point.

Parents of 12–17‐year‐olds mentioned adolescents' not valuing their opinions more (27.3%) compared to parents of 7–11‐year‐olds (8.3%).

#### Younger children need shorter bits of information

Some parents discussed needing to have shorter conversations with younger children and longer conversations with adolescents. Parents also reported an emphasis on solution‐based topics for younger children which included action steps that they could take, as opposed to conversations where there is not an easy solution. For example,

Well, when you talk to younger kids… I've found it's better to give short snippets of things… definitely it can be overwhelming for them to have a long discussion about something that's painful… and that there doesn't seem to be an immediate solution that they can fix it.

Parents of 7–11‐year‐olds mentioned this idea that younger children need shorter bits of information more (16.7%) compared to parents of 12–17‐year‐olds (9.1%).

### Domain Four: White Identity and Privilege

**FIGURE 4:**
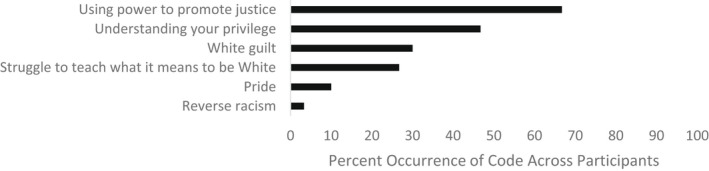
Domain IV: White identity and privilege.

To address the final research question involving what parents teach their children about being White, participants reported various opinions and discussions surrounding White identity and privilege, which were coded into six themes, including using power to promote justice, understanding privilege, White guilt, a struggle to define and teach the meaning of Whiteness, and reverse racism (see Figure 4).

#### Using power to promote justice

Many parents reported wanting their children to use their White privilege in order to “help others” and be an ally. For some, the focus was on their children to “help people of color,” whereas for others the focus was to change the systems of oppression. For example, “I always like them to understand that they have this White privilege that is a very powerful tool that they can use to change the system… and just try to educate other people.” Other parents reported emphasizing the duty of White individuals to speak up because of the White privilege that the hold. Finally, some parents emphasized wanting their child to have an active role in issues surrounding equity and inclusion, including speaking up when they hear other individuals making racist comments and participating in activism such as protests and political organizations.

#### Understanding your privilege

Some parents reported having conversations with their children regarding various aspects of privilege. For example, one parent stated “I'm raising two White males…I told them that there are definitely things that they have privilege to that other people don't… that they have it a lot easier than a lot of other people do.” Several other parents emphasized how White individuals are the majority, which grants the privilege of not being the only person in a room who looks like themselves. Additionally, several parents identified not engaging in conversations about racial privilege, but instead discussing economic privilege with their children. For example, “I want them to be aware that… the socioeconomic class that we live in… is not necessarily the norm… and to not take that for granted and not judge people because they're in different walks of life.”

#### White guilt

Some parents identified concerns of not wanting their children to feel bad or guilty because they are White. For example,

…I just don't want them to think that they're less… or bad people because they're White… I'm kind of afraid that that's where our country is headed a little bit with school curriculums and things like that… that we're like inherently… evil somehow because we have white skin…

Other parents reported feelings guilty due to their own privilege. For example, “It's really tiring. It's emotionally draining… It's like, look at us to have the privilege to walk away from a draining conversation and not have to think about it… to only be drained momentarily.”

#### Struggle to teach what it means to be White

Some parents struggled to identify what they would like to teach their children about what it means to be White. For example, “I… don't know if I have a good answer about that like if they ever asked me like… ‘What does it mean to be white?’… it's just how I was born.” Another parent mentioned,

I'm not sure that there's anything that I want to teach them about being White… I don't know I mean I can't think of how I would teach that…. I guess maybe there's conversations to be had there about growing up with privilege and how we can help others but… I don't know.

Other parents also struggled to identify a clear meaning for what message they would like to provide. For example, one parent reported telling their children,

“Don't just think you can slide by in life and not have to work hard for anything… you're just as low as anybody else or just as high as anybody else whichever way you want to look at it… like everyone is equal. So don't think that you're better than somebody” I guess is the best way to put it… “just because of the color of your skin.”

#### Pride

Very few parents referenced pride, wanting their children to feel proud but struggling to find things to be proud of. For example, as one mother explained:

My husband has said a lot about this like “jeez it's hard to be proud about very much right now” … I know he [son] knows our lineage, but I do not think any of us in our household right now are very proud to be anything other than an ally to people who do not have the privilege like we have.

#### Reverse racism

Only one participant brought up the idea that they believe racism can go in both directions. For example, “…sometimes I feel like maybe… it's also racism the way that White people are portrayed as being racists and bigots and hateful…. Sometimes I think racism can work both directions a little bit.”

## DISCUSSION

Our findings demonstrate multiple perspectives in a sample of White parents of White 7–17‐year‐olds residing in the Midwest with respect to the content of conversations addressing race, factors influencing socialization practices, and White identity. These findings need to be understood in the historic context of a racialized system and recent racial justice movements that have been at the forefront of public discourse in the United States.

Previous research on WRS has identified color‐conscious and color‐blind racial approaches as two main types of racial socialization (Loyd & Gaither, 2018). Consistent with the mixed messages evident in our interview data, Abaied and Perry (2021) demonstrated that a third of their sample of White parents endorsed both color‐conscious and color‐blind racial socialization approaches. Although some participants in this study mentioned either of these two viewpoints, many participants did not fall into this binary. For example, some participants indicated a combination of these approaches, such as talking to their children about historic or current racial tensions at a societal level while employing what Neville et al. (2013) refer to as color‐evasive beliefs (e.g., not wanting their child to see color or teaching their children broad morals such as treat everyone equal without reference to racial inequities) in individual interactions. Overall, color evasive beliefs (reference to fair and equal treatment of all without specifying race) were more common (endorsed by 76.7%) than specific references to color‐blind racial views (not wanting the child to see color, 13.3%). This is not surprising given that color‐blind racial socialization perspectives are often implied through the avoidance of specific race‐related conversations, such as focusing on broad morals.

Another example of mixed messages in our data can be found in parents' discussion of the BLM movement. Although a majority of the parents expressed support for the movement, nearly half of the parents went on to condemn rioting or looting. By discussing “rioting,” the attention is diverted away from the key issue of why the protests are taking place and what needs to be done to achieve racial justice. Additionally, few parents reported not publicly expressing support for BLM due to not wanting to upset friends or family who are police officers, or expressed hesitation to discuss these issues in detail with their children due to worries that their child will believe that all police officers are bad or dangerous. Overall, these findings highlight that White parents' perspectives on race are multi‐layered, and often contain mixed messages that cannot be clearly captured by racially color‐blind versus color‐conscious dichotomy.

### Concerns and Considerations in Having Conversations About Race

A vast majority of parents in our sample reported that conversations about race are important for their child to learn about diversity and to make the world a “better place.” Findings from previous research are mixed with earlier work showing that White parents do not believe that it is important to discuss topics related to race with their children (Katz, 2003), whereas recent work has shown that White parents consider these conversations as important (Vittrup, 2018). Our findings add to this literature by demonstrating not only that White parents consider conversations about race to be important, but also what specific benefits they perceive of engaging in these conversations. As Underhill (2019) describes, White middle‐class parents' focus on exposure to diversity may be motivated by a desire to move away from past practices of racial segregation, promote interracial comfort, and potentially enable social change in individual interactions with people of color. However, as Underhill argues, exposure to diversity without acknowledgment of racial inequities is unlikely to promote awareness and understanding of the experiences of people of color and actually enable social change.

Although conversations about race were considered to be important and a majority of parents reported benefits of such conversations, some parents in our sample expressed concerns about having such conversations for various reasons. Consistent with existing research (Hagerman, 2014; Hamm, 2001; Vittrup, 2018), parents reported both child‐centered concerns (a desire to protect the child due to child's age, maturity, or personal characteristics, fears that their child may misinterpret or overemphasize the importance of race, not wanting the child to see color) and parent‐centered concerns (parents' own discomfort or lack of knowledge). Abaied and Perry (2021) summarized White parents' child‐centered concerns as parents' desire to shield their child from negativity or from “adult matters,” and Underhill (2018) describes this as White parents' desire to create a “protected, worry‐free childhood.” White parents' desire to protect their children or to not be knowledgeable or comfortable in discussing race related matters reflects underlying White privilege: White parents have the privilege to not know or to shield their children from learning about mistreatment of people of color, a privilege not shared by people of color.

### What Gets Talked About, and What Does Not

White parents in our sample reported talking about historic contexts (i.e., slavery, Native American history, civil rights movement), as well as contemporary publicized events (BLM, COVID‐19 anti‐Asian rhetoric, immigration‐related issues) more than discussing what people of color experience in day‐to‐day life such as systemic racism and racial microaggressions. With regard to history, parents reported engaging in conversations about topics their child was learning in school, as well as visiting museums or memorials. Discussing historic mistreatment of people of color is critical so that the children can understand the longstanding oppression in the US society (Hughes et al., 2007). However, it is important to ensure that a focus on history does not inadvertently communicate that mistreatment and oppression only occurred in the past.

Helping youth to understand contemporary events and movements, as well as media coverage of those events is critical and a small proportion of parents in our sample explicitly reported participating in social justice movements, whereas others expressed concerns regarding rioting, looting, and destruction of property, rather than focusing on the system of oppression that contributes to acts of police brutality. Conversations about historic or contemporary events can provide meaningful learning opportunities for children if they involve discussions of inherent racial inequities and systematic oppression that contribute to these situations (Hughes et al., 2007). Without acknowledgment of the role of power and inequities, cursory references to historic or current events are unlikely to enable White children to understand the mistreatment of people of color. In our data, parents' responses ranged from brief conversations that lacked nuanced and avoided discussion of power to responses that acknowledged the past and present racism and racial inequities.

In addition to broader societal movements, discussion of current systems of oppression that includes less publicized experiences in one's own communities is also critical, and these were less frequent in our data. For example, there were significantly fewer references to conversations around systematic racism taking place in schools, workplaces, healthcare systems that is not highly publicized in the media, along with daily experiences of racial microaggressions that contribute to added stress for people of color. Although a majority of parents in our sample did not explicitly deemphasize disproportionate distribution of power, relatively fewer references to systemic racism and microaggressions, and not fully discussing the role of racial inequities in conversations about historic or current events may represent what Neville et al. (2013) refer to as power evasion beliefs where the role of power is minimized or denied. Limited focus on systemic racism and racial microaggressions represents a major gap in WRS efforts that needs to be addressed through higher awareness among White parents of the importance of discussing these experiences, along with educational programs that focus on developing parents' skills for effectively talking to their children about these aspects.

In a little over three‐fourths of the interviews, references were made to broad morals such as “treat everyone equal” and bullying without specifying race. Here, parents discussed that they focused on teaching their children values like “treat others the way you want to be treated” and “be nice to others,” “stand up for others,” or “be a friend to everyone” but often did not explicitly mention race‐based bullying. Our findings are consistent with prior research (Zucker & Patterson, 2018), which found that even in situations where race was salient, many White parents chose to not directly or explicitly discuss race. These responses indicate color‐evasion beliefs, where people report viewing each person as an individual and endorse fairness toward everyone (Neville et al., 2013).

### Teaching What it Means to be White

In over half of our interviews, White parents described encouraging their children to use their power or privilege to “help others” and promote justice. Some parents also emphasized to their children that as White individuals, they do not truly understand the experiences of people of color. When asked about what it means to be White in America, some parents reported not having thought about it prior to the interview, and struggled to identify what messages they would like to convey to their children. This finding is consistent with previous research demonstrating that White individuals often do not think of themselves in terms of their racial identity, and therefore, the White experience is often viewed as the “standard” (e.g., Herman, 2004; Lewis, 2004).

A majority of parents in our sample discussed encouraging their children to understand their privilege and to use it to promote justice by helping others, speaking up, and participating in activism. It is important to consider this finding within the context of relatively fewer references in our data regarding conversations about systemic racism or microaggressions. If conversations about understanding one's privilege are not grounded in inherent power differences embedded in institutional racism (Neville et al., 2013), they are unlikely to help achieve racial justice. In order to promote true allyship, White individuals must demonstrate a nuanced understanding of institutional racism and privilege, as well as engage in practices such as self‐reflection, disrupt racism at micro and macro levels, promote equity, participate in coalition building with people of color, and encounter resistance from other White individuals (Spanierman & Smith, 2017). Parents in this study referenced components of allyship such as self‐reflection and promoting justice, but not much about challenging institutional racism. Some parents also described desires for their children to “help people of color.” When engaging in such conversations of helping people of color, it is important for White parents to reflect on the most optimal ways to help, for example, helping by challenging systems of White supremacy. This type of allyship that disrupts systems of oppression are indeed helpful compared to a paternalistic attempt at allyship that involves helping people of color survive within White supremacy (Spanierman & Smith, 2017).

Parents also discussed emotions such as guilt in reference to being White. In line with research by Gillen‐O'Neel, Huynh, Hazelbaker, and Harrison (2021), a common concern included a desire to avoid making their children feel guilty because they are White. For some White parents this desire may lead to having race‐related conversations less frequently with their children, whereas other parents may engage in these conversations despite these concerns. Additional research may seek to clarify what specific aspects contribute to a parent believing the benefits outweigh the concerns when discussing racism with their children. It is important that White parents focus on teaching their children to take responsibility for historic and contemporary systems of oppression and engage in allyship and advocacy rather than focus on avoiding guilt. Additionally, parents may instead strive to promote White empathy, or the ability to empathize with people of color in experiences of racism (Chao, Wei, Spanierman, Longo, & Northart, 2015). Parents may also incorporate White role models who have challenged these systems of oppression, served as agents who engaged in advocacy and social change, who may serve as sources of pride for the White community.

### Developmental Differences

Parents reported engaging in more complex and longer conversations with adolescents compared to younger children because they believed that adolescents are capable of understanding and emotionally managing difficult topics that are often involved in talking about race relations. However, parents differed with respect to what they considered to be an appropriate age to have conversations about race. For example, one parent reported that her 7‐year‐old was old enough to better understand concepts such as oppression and police brutality, while another parent reported that her 12‐year‐old was not yet ready for specific details regarding oppression and police brutality. White parents in previous studies have expressed similar concerns that their children between the ages of 8 and 12 are too young for conversations related to race (Abaied & Perry, 2021). Clearly, White parents' perceptions of the child's readiness and maturity are critical in their decisions regarding racial socialization.

Similar to previous research (Hagerman, 2014; Loyd & Gaither, 2018), parents in our sample recognized that adolescents have direct access to current events, public conversations regarding movements such as BLM through media and peers, and that adolescents' perspectives may or may not align with those of parents. Several parents of adolescents reported conversations about current events being initiated by their adolescents, and in the process, they may motivate parents to learn more about social movements. In this way, WRS processes may be bidirectional for parents and adolescents where they learn from one another.

### Limitations and Future Research Directions

This study included a sample of White parents (predominantly mothers, who were largely middle class or above and highly educated), who had access to social media, and who were residing in the Midwestern US. It is likely that these parents were particularly exposed to media coverage and public conversations regarding racial injustice, which likely contributed to their narratives. Further, parents in this sample may have more economic resources such as access to books, being able to travel to and visit museums, or access to extracurricular programs for their children than parents from lower socioeconomic class backgrounds (Hagerman, 2017). Although there were no notable differences between the mothers and one father participant in this sample, the lack of additional fathers limits the ability to draw meaningful conclusions regarding potential gender differences in WRS. There is also a possibility of self‐selection such that White parents who feel less comfortable discussing topics related to race, or hold beliefs that do not acknowledge racial inequity may not have chosen to participate in this study. Thus, although the findings help us understand WRS among White middle‐class mothers residing in the Midwest, they may not be applicable to White fathers, or parents from lower socioeconomic backgrounds.

The major aim of a qualitative study is to derive understanding of a phenomenon deeply rather than produce generalizable information. Future research should expand upon these findings with White parents of various other social identities, including and parents from other geographic regions, socioeconomic class, and education level. In this study, two participants reported also having adopted children of color in addition to their White children. Although this was not a focus of the current study, a separate body of literature has examined the racial socialization practices of White parents raising biracial children and adopted children of color (Umaña‐Taylor & Hill, 2020). However, to the best of our knowledge, no existing study has examined how adopted or biracial children influence White parents' racial socialization of their White children. Additionally, future research may investigate racial socialization among White mothers and White fathers as racial socialization or beliefs may vary by gender (Spanierman, Beard, & Todd, 2012), as well as trans and gender nonbinary parents, and children with varying gender identity to explore intersecting influences of race and gender privilege or oppression. Although the semi‐structured interview format allowed us to gather White parents' perspectives on WRS in their own words, because this format does not afford anonymity to the participants, social desirability concerns may have been enhanced, contributing to what participants chose to share. Future research may expand upon these findings through quantitative and mixed methods approaches to empirically assess WRS and incorporate child/adolescent report of parental strategies.

Despite limitations, this study adds to existing literature by providing rich descriptions of the range of topics that White parents discuss pertaining to race and privilege with their children, as well as factors parents consider when deciding whether or not to have these conversations. These findings may contribute to refining theoretical frameworks of WRS, and to the development of survey measures that assess WRS. The findings may also inform educational resources for White parents who are interested in engaging in racial socialization but feel uncomfortable or unprepared for how to effectively engage in discussing difficult topics. Although exposing children to diversity (Underhill, 2019) and helping them appreciate differences is a critical building block, WRS needs to move beyond this to a systematic effort that incorporates conversations about the role of disproportionate power and privilege of White individuals that has historically contributed to racial inequities and racial oppression, which continues to this day, day‐to‐day experiences of people of color (racial microaggressions, systemic racism across settings) and their impact on health, well‐being, and quality of life, and the critical responsibility of White youth to engage in allyship and advocacy to promote social change. Enabling White parents is essential in preparing the next generation of White Americans who are able to serve as allies and advocates for people of color, moving a step closer to achieving racial justice.

## ACKNOWLEDGEMENTS

We wish to extend our appreciation to the research participants of this study.

## References

[jora12782-bib-0001] Abaied, J. L. , & Perry, S. P. (2021). Socialization of racial ideology by white parents. Cultural diversity and ethnic minority psychology, 27, 431–440. 10.1037/cdp0000454 33914582

[jora12782-bib-0002] Aboud, F. E. , Mendelson, M. , & Purdy, K. (2003). Cross‐race peer relations and friendship quality. International Journal of Behavioral Development, 27, 165–173.

[jora12782-bib-0003] Bigler, R. S. , & Liben, L. S. (2007). Developmental intergroup theory: Explaining and reducing children's social stereotyping and prejudice. Current Directions in Psychological Science, 16, 162–166.

[jora12782-bib-0004] Bigler, R. S. , & Wright, Y. F. (2014). Reading, writing, arithmetic, and racism? Risks and benefits to teaching children about intergroup biases. Child Development Perspectives, 8, 18–23.

[jora12782-bib-0005] Bonilla‐Silva, E. (1997). Rethinking racism: Toward a structural interpretation. American Sociological Review, 62(3), 465–480.

[jora12782-bib-0006] Braun, V. , & Clarke, V. (2006). Using thematic analysis in psychology. Qualitative Research in Psychology, 3(2), 77–101.

[jora12782-bib-0007] Chao, R. C. L. , Wei, M. , Spanierman, L. , Longo, J. , & Northart, D. (2015). White racial attitudes and white empathy: The moderation of openness to diversity. The Counseling Psychologist, 43(1), 94–120.

[jora12782-bib-0008] Edwards, J. F. (2017). Color‐blind racial attitudes: Microaggressions in the context of racism and white privilege. Administrative Issues Journal, 7(1), 2.

[jora12782-bib-0009] Feagin, J. (2013). Systemic racism: A theory of oppression. Routledge.

[jora12782-bib-0010] Gillen‐O'Neel, C. , Huynh, V. W. , Hazelbaker, T. , & Harrison, A. (2021). From kindness and diversity to justice and action: White parents' ethnic‐racial socialization goals. Journal of Family Issues, 43(4), 1–30.

[jora12782-bib-0011] Hagerman, M. (2014). White families and race: Colour‐blind and colour‐conscious approaches to white racial socialization. Ethnic and Racial Studies, 37, 2598–2614.

[jora12782-bib-0012] Hagerman, M. (2017). White racial socialization: Progressive fathers on raising “antiracist” children. Journal of Marriage and Family, 79, 60–74.

[jora12782-bib-0013] Hamm, J. V. (2001). Barriers and bridges to positive cross‐ethnic relations: African American and white parent socialization beliefs and practices. Youth Society, 33, 62–98.

[jora12782-bib-0014] Herman, M. (2004). Forced to choose: Some determinants of racial identification in multiracial adolescents. Child Development, 75, 730–748. 15144483 10.1111/j.1467-8624.2004.00703.x

[jora12782-bib-0015] Hughes, D. , Rodriguez, J. , Smith, E. P. , Johnson, D. J. , Stevenson, H. C. , & Spicer, P. (2006). Parents' racial/ethnic socialization practices: A review of research and agenda for future study. Developmental Psychology, 42, 747–770. 16953684 10.1037/0012-1649.42.5.747

[jora12782-bib-0016] Hughes, J. M. , Bigler, R. S. , & Levy, S. R. (2007). Consequences of learning about historical racism among European American and African American children. Child Development, 78, 1689–1705. 17988315 10.1111/j.1467-8624.2007.01096.x

[jora12782-bib-0017] Hunter, A. G. , Friend, C. A. , Williams‐Wheeler, M. , & Fletcher, A. C. (2012). Race, class, and religious differences in the social networks of children and their parents. Youth Society, 44, 450–475.

[jora12782-bib-0018] Johnson, J. , Rush, S. , & Feagin, J. (2000). Doing anti‐racism and making a non‐racist society. Contemporary Sociology, 29, 95–109.

[jora12782-bib-0019] Katz, P. A. (2003). Racists or tolerant multiculturalists? How do they begin? American Psychologist, 58, 897–909. 14609382 10.1037/0003-066X.58.11.897b

[jora12782-bib-0020] Lesane‐Brown, C. S. , Spatzier, A. , & Tobin, M. (2010). Variability in the inter‐group attitudes of white children: What we can learn from their ethnic identity labels. Social Development, 19, 758–778.

[jora12782-bib-0021] Levitt, H. M. , Bamberg, M. , Creswell, J. W. , Frost, D. M. , Josselson, R. , & Suárez‐Orozco, C. (2018). Journal article reporting standards for qualitative primary, qualitative meta‐analytic, and mixed methods research in psychology: The APA publications and communications board task force report. American Psychologist, 73(1), 26–46. 29345485 10.1037/amp0000151

[jora12782-bib-0022] Lewis, A. E. (2004). “What group?” studying whites and whiteness in the era of color‐blindness. Sociological Theory, 22, 623–646.

[jora12782-bib-0023] Lord, J. , Schnarr, A. , & Hutchison, P. (1987). The voice of the people: Qualitative research and the needs of consumers. Canadian Journal of Community Mental Health, 6(2), 25–36.

[jora12782-bib-0024] Loyd, A. B. , & Gaither, S. E. (2018). Racial/ethnic socialization for white youth: What we know and future directions. Journal of Applied Developmental Psychology, 59, 54–64.

[jora12782-bib-0025] Morrow, S. L. (2005). Quality and trustworthiness in qualitative research in counseling psychology. Journal of Counseling Psychology, 52(2), 250–260.

[jora12782-bib-0026] Neblett, E. W. , Rivas‐Drake, D. , & Umaña‐Taylor, A. J. (2012). The promise of racial protective factors in promoting ethnic minority youth development. Child Development Perspectives, 6, 295–303.

[jora12782-bib-0027] Nesdale, D. (2013). Social acumen: Its role in constructing group identity and attitudes. In M. R. Banaji & S. A. Gelman (Eds.), Navigating the social world: What infants, children, and other species can teach us (pp. 323–326). Oxford University Press.

[jora12782-bib-0028] Neville, H. A. , Awad, G. H. , Brooks, J. E. , Flores, M. P. , & Bluemel, J. (2013). Color‐blind racial ideology: Theory, training, and measurement implications in psychology. American Psychologist, 68, 455–466. 24016116 10.1037/a0033282

[jora12782-bib-0029] O'Connor, C. , & Joffe, H. (2020). Intercoder reliability in qualitative research: Debates and practical guidelines. International Journal of Qualitative Methods, 19, 1–13.

[jora12782-bib-0030] Pahlke, E. , Bigler, R. S. , & Suizzo, M. (2012). Relations between colorblind socialization and children's racial bias: Evidence from European American mothers and their preschool children. Child Development, 83, 1164–1179. 22537347 10.1111/j.1467-8624.2012.01770.x

[jora12782-bib-0031] Quintana, S. M. (1998). Children's developmental understanding of ethnicity and race. Applied and Preventative Psychology, 7, 27–45.

[jora12782-bib-0032] Quintana, S. M. , & McKown, C. (Eds.). (2008). Handbook of race, racism, and the developing child. John Wiley & Sons.

[jora12782-bib-0033] Shaw, A. , Montinari, N. , Piovesan, M. , Olson, K. R. , Gino, F. , & Norton, M. I. (2014). Children develop a veil of fairness. Journal of Experimental Psychology: General, 143(1), 363–375. 23317084 10.1037/a0031247

[jora12782-bib-0034] Spanierman, L. B. , Beard, J. C. , & Todd, N. R. (2012). White men's fears, white women's tears: Examining gender differences in racial affect types. Sex Roles, 67, 174–186.

[jora12782-bib-0035] Spanierman, L. B. , & Smith, L. (2017). Roles and responsibilities of white allies: Implications for research, teaching, and practice. The Counseling Psychologist, 45(5), 606–617.

[jora12782-bib-0036] Steinberg, L. , & Morris, A. S. (2001). Adolescent development. Annual Review of Psychology, 52, 83–110. 10.1146/annurev.psych.52.1.83 11148300

[jora12782-bib-0037] Sue, D. W. , & Spanierman, L. B. (2020). Microaggressions in everyday life (2nd ed.). John Wiley & Sons.

[jora12782-bib-0038] Tessler, H. , Choi, M. , & Kao, G. (2020). The anxiety of being Asian American: Hate crimes and negative biases during the COVID‐19 pandemic. American Journal of Criminal Justice, 45, 636–646. 32837158 10.1007/s12103-020-09541-5 PMC7286555

[jora12782-bib-0039] Umaña‐Taylor, A. J. , & Hill, N. E. (2020). Ethnic‐racial socialization in the family: A decade's advance on precursors and outcomes. Journal of Marriage and Family, 82, 244–271.

[jora12782-bib-0040] Underhill, M. R. (2018). Parenting during Ferguson: Making sense of white parents' silence. Ethnic and Racial Studies, 41(11), 1934–1951.

[jora12782-bib-0041] Underhill, M. R. (2019). “Diversity is important to me”: White parents and exposure‐to‐diversity parenting practices. Sociology of Race and Ethnicity, 5(4), 486–499.

[jora12782-bib-0042] Verkuyten, M. , Kinket, B. , & van der Wielen, C. (1997). Preadolescents' understanding of ethnic discrimination. The Journal of Genetic Psychology, 158(1), 97–112. 9120410 10.1080/00221329709596655

[jora12782-bib-0043] Vittrup, B. (2018). Color blind or color conscious? White American mothers' approaches to racial socialization. Journal of Family Issues, 39, 668–692.

[jora12782-bib-0044] Zucker, J. K. , & Patterson, M. M. (2018). Racial socialization practices among white American parents: Relations to racial attitudes, racial identity, and school diversity. Journal of Family Issues, 39(16), 3903–3930.

